# PW03-011 – New Behçet's loci and gene-gene interactions

**DOI:** 10.1186/1546-0096-11-S1-A237

**Published:** 2013-11-08

**Authors:** EF Remmers, Y Kirino, G Bertsias, MJ Ombrello, G Wood, Y Ishigatsubo, N Mizuki, I Tugal-Tutkun, E Seyahi, Y Ozyazgan, DL Kastner, A Gül

**Affiliations:** 1 Inflammatory Dis Section/MGB, NHGRI, Bethesda, USA; 2 Int Med and Clin Immunol, Yokahama City University, Yokohama, Japan; 3 Ophthalmology and Visual Sci, Yokahama City University, Yokohama, Japan; 4 Istanbul Faculty of Medicine, Istanbul University, Istanbul, Turkey; 5 Cerrahpaşa Faculty of Medicine, Istanbul University, Istanbul, Turkey

## Introduction

We previously identified disease-associated common variants in *IL10* and *IL23R*, as well as HLA-B*51, in a Behçet’s disease (BD) genome-wide association study (GWAS) performed with 311,459 SNPs in 1,215 cases and 1,278 controls from Turkey, but the disease-associated variants in these genes do not fully account for the estimated genetic contribution to disease risk.

## Objectives

To discover novel common BD susceptibility variants and to evaluate disease loci for evidence of gene-gene interactions.

## Methods

We used the Turkish collection GWAS genotypes to impute genotypes of 779,000 markers in the GWAS subjects and then evaluated the imputed markers for disease association. We also searched for new disease associated loci by analyzing patients with uveitis and by specifying different genetic models. We replicated the new BD loci in additional Turkish samples (838 cases, 630 controls) and if polymorphic, in Japanese samples (612 cases, 740 controls). Gene-gene interactions were evaluated by testing the significance of a multiplicative interaction term in a logistic regression model.

## Results

Imputation implicated three new BD susceptibility loci (*CCR1*, *STAT4*, and *KLRC4*). Validation, fine-mapping, and replication confirmed these associations and meta-analyses identified variants with genome-wide significance (p<5x10^-8^) in each. The variants in *CCR1*, CC-chemokine receptor 1, and *STAT4*, signal transducer and activator of transcription 4, were associated with gene expression differences. PBMCs with the disease-associated *CCR1* variant exhibited reduced migration to the CCR1 ligand, MIP1a. Two disease-associated variants in *KLRC4*, which encodes an NK receptor family member, encoded missense changes (I29S and N104S). The BD-associated HLA-B*51 haplotype includes *MICA*, an NK receptor ligand. A statistically significant interaction (p=0.03) was identified between HLA-B*51 (presumably tagging *MICA* variation) and *KLRC4* N104S. Analysis of BD patients with uveitis identified two non-synonymous variants (D575N and R725Q) in *ERAP1* that recessively conferred BD risk (p=4.7x10^−11^). ERAP1 is an endoplasmic reticulum-expressed aminopeptidase that trims peptides and loads them onto MHC Class I. We found strong evidence for an interaction between the BD-associated Class I allele HLA-B*51 and ERAP1 genotype (p=9x10^−4^).

## Conclusion

This study identified four new genetic loci (*CCR1*, *STAT4*, *KLRC4*, and *ERAP1*) and two gene-gene interactions (*ERAP1* with HLA-B*51 and *KLRC4* with HLA-B*51, presumably via its LD with *MICA*) that contribute to BD susceptibility. Shared genetic associations of MHC Class I, *IL23R*, and *ERAP1*, and the strong interactive effect of the disease-associated Class I allele and *ERAP1* support an emerging concept that BD, ankylosing spondylitis, and psoriasis share pathogenic mechanisms.

## Disclosure of interest

None declared

